# Connaissances du paludisme et attitudes préventives des voyageurs tunisiens vers les zones d’endémie palustre

**DOI:** 10.11604/pamj.2022.41.223.28696

**Published:** 2022-03-17

**Authors:** Sonia Kechaou-Cherif, Mohamed Hsairi, Aida Bouratbine, Alia Benkahla, Samy Khoufi, Karim Aoun

**Affiliations:** 1 Service des Vaccinations Internationales et Antirabiques, Institut Pasteur de Tunis, 13, place Pasteur, BP 74,1002 Tunis Belvédère, Tunisie,; 2 Laboratoire de BioInformatique, bioMathématiques, bioStatistique, LR 16-IPT-09, Institut Pasteur de Tunis, Université Tunis El Manar, Tunis, Tunisie,; 3 Service d´Epidémiologie, Institut Salah Azaiez de Carcinologie, Université Tunis El Manar, Tunis, Tunisie,; 4 Laboratoire de Parasitologie Médicale, Biotechnologies et Biomolécules, LR 20-IPT-06, Institut Pasteur de Tunis, Université Tunis El Manar, Tunis, Tunisie,; 5 Laboratoire d´Epidémiologie et d´Ecologie Parasitaires, Institut Pasteur de Tunis, Tunis, Tunisie

**Keywords:** Paludisme, prévention, connaissance, attitudes, voyageurs, Tunisie, Malaria, prevention, knowledge, attitudes, travellers, Tunisia

## Abstract

**Introduction:**

la connaissance du paludisme, de ses facteurs de risque et de sa prophylaxie par les voyageurs se rendant en zones d'endémie est fondamentale. Elle permet d'éviter les cas graves de la maladie et de prévenir la reprise de la transmission dans des pays indemnes. Cette étude vise à évaluer les connaissances des voyageurs tunisiens quant au paludisme, sa transmission, sa prévention et leur adhésion aux mesures prophylactiques.

**Méthodes:**

une enquête basée sur deux questionnaires anonymes (pré et post voyage) a été menée auprès d´adultes voyageant vers les pays endémiques. Le 1^er^ questionnaire a été suivi d´un entretien médical centré sur les risques encourus et les mesures prophylactiques.

**Résultats:**

au total, 289 voyageurs ont été recrutés dont 99% (n=286) se déplaçaient en Afrique subsaharienne et 84,4% (n=244) pour des raisons professionnelles. Leur moyenne d´âge était de 42,3 ans (± 12,5). La proportion des hommes était de 74,4% (n=218). En pré-voyage, 53,3% (n=154) avaient conscience d´un risque de paludisme et 28% (n=81) connaissaient les modalités de transmission. La recommandation d´une chimioprophylaxie n´était connue que par 62,3% (n=180) et 43,6% (n=126) comptaient en prendre (p < 0,01). Une meilleure adhésion aux mesures de protection dont la chimioprophylaxie a été notée en post-voyage avec des attitudes qualifiées de bonnes ou excellentes de 64,2% (n=95) au retour contre 23,7% (n=35) avant l´entretien (< 0,001).

**Conclusion:**

les connaissances des voyageurs tunisiens à propos du paludisme sont insuffisantes. Un renforcement de l´information s´impose particulièrement par le biais de consultations spécialisés dont l´utilité a été démontrée.

## Abstract (en)

**Introduction:**

travellers to endemic areas must know malaria, its risk factors and prophylactic measures. This can help to avoid severe cases of malaria and to prevent transmission in countries that are malaria-free. The purpose of this study is to assess Tunisian travellers´ knowledge about malaria, its transmission and prevention and their adherence to prophylactic measures.

**Methods:**

we conducted a survey based on two anonymous questionnaires (pre- and post-trip) among adults travelling to endemic countries. The 1st questionnaire was followed by a medical interview focusing on level of risk and recommended prophylactic measures.

**Results:**

two hundred and eighty-nine travellers were recruited. They mainly moved within sub-Saharan Africa (99%) for professional reasons (84,4%). The average age of subjects was 42.3 years and sex ratio (male/female) was 3.1. Prior to departure, only 53.3% of subjects were aware of the risk of malaria, and only 28% gave correct answers about modes of transmission. Recommendations for chemoprophylaxis were only known by 62.3% of subjects and only 43.6% intended to use chemoprophylaxis (p < 0.01). Better adherence to protective measures, including chemoprophylaxis, was reported after the trip, with attitudes qualified as good or excellent by 64.2% on return against 23.7% before the interview (<0.001).

**Conclusion:**

Tunisian travellers knowledge of malaria is insufficient. Strengthening information through specialized consultations (whose usefulness has been demonstrated) is required.

## Introduction

Le paludisme a été éliminé de Tunisie en 1979; depuis, seuls des cas importés sont enregistrés [1]. L´incidence de ces cas est en hausse ces 10 dernières années (environ 100/an) ce qui augmente la morbidité de la maladie et fait ressurgir le risque de reprise d´une transmission autochtone surtout que les anophèles vecteurs sont encore présents à travers le pays [2,3]. Dans ce contexte, la maîtrise des facteurs de risque de la maladie et la promotion des mesures individuelles de prévention pour les voyageurs exposés s´avèrent un volet fondamental du programme national de contrôle du paludisme et de maintien de son élimination. En effet, les voyages en zones d´endémie sont de nos jours les principaux pourvoyeurs de cas de paludisme chez les ressortissants et résidants des pays indemnes. Ainsi, une méta-analyse récente à montré que le paludisme représente la première étiologie de fièvre (22%) au retour de voyages [4,5]. Les efforts de prévention devraient surtout concerner les voyages vers les pays d´Afrique subsaharienne (Afrique SS) où sont enregistrées les incidences les plus élevées de la maladie, et ce, surtout que les échanges économiques de la Tunisie avec ces pays sont en nette progression [5]. Ceci se confirme dans les données françaises qui révèlent qu´environ 95% des cas de la maladie font suite à un séjour en Afrique SS [6]. Pour cela, un bon système d´information autour de la maladie, sa transmission et sa prophylaxie semble incontournable. L´amélioration et le développement d´un tel système passe nécessairement par une évaluation préalable de l´état des lieux. Ainsi, le but de cette étude était d´estimer le niveau de connaissance du paludisme et de ses modalités de transmission et de prévention chez les voyageurs tunisiens en partance vers les pays d´endémie palustre ainsi que leur adhésion aux différentes méthodes de prophylaxie proposées et disponibles.

## Méthodes

### Cadre et conception de l´étude

Une enquête a été menée au service des vaccinations internationales et antirabiques de l´institut Pasteur de Tunis (IPT) où les voyageurs se présentent pour faire des vaccinations obligatoires conformément au règlement sanitaire international et aux recommandations nationales en la matière [7]. L´enquête s´est déroulée sur une période d´un mois, du 14 septembre au 13 octobre 2017, en marge des séances de vaccinations internationales. Les voyageurs qui ont accepté de participer à l´enquête ont reçu une lettre d´information détaillant l´étude ainsi qu´un formulaire de consentement et le questionnaire relatif à l´enquête. Une fois le formulaire de consentement signé et le questionnaire rempli, les voyageurs ont reçu via un médecin des informations quant au paludisme, ses modalités de transmission, le risque de contamination encouru lors de leur voyage et les différentes mesures prophylactiques possibles. A la fin de l´entretien, il leur a été proposé de nous communiquer leur contact téléphonique afin de répondre, s´ils le souhaitent, à un 2^e^ questionnaire au retour de leurs voyages. Pour ceux qui ont accepté, un registre contenant anonymement les numéros de téléphone a été tenu. Un mois après la date de retour de voyage, ces derniers ont été rappelés et ont eu un 2^e^ entretien au téléphone avec un médecin. Les 2 questionnaires étaient anonymes, codés et ne comportaient pas de données personnelles. Toutes les questions étaient simples et courtes. Certaines sont à choix multiples et d´autres à réponse courte.

**La population étudiée:** elle est représentée par les voyageurs âgés de plus de 18 ans, en partance vers un pays d´endémie palustre, qui ont consulté pour des vaccinations internationales durant la période de l´enquête. Seuls les voyageurs ayant donné leur consentement éclairé pour participer à l´étude ont été inclus. Ceux ayant refusé n´ont pas été retenus.

**Recueil des données:** les données ont été collectées à partir des deux questionnaires proposés en pré et post voyage. Le 1^er^ questionnaire de pré-voyage a été proposé en deux versions au choix, l´une en langue arabe et l´autre en langue française. Il s´agissait d´un questionnaire en auto-évaluation comportant 18 questions qui ont permis de recueillir des données concernant le voyageur (l´âge, le sexe, le niveau d´instruction, voyages antérieurs en zone d´endémie palustre), ses sources d´information concernant le paludisme, son voyage actuel (motif, destination, dates, conditions d´hébergement, déplacements éventuels sur place), l´évaluation de ses connaissances sur le paludisme, ses modalités de transmission et sa prophylaxie et enfin sa perception du risque et sa prédisposition à l'adhésion aux différentes mesures de prophylaxie anti-palustre. Le 2^e^ questionnaire de post-voyage comptait 3 questions. Il était sous forme d´entretien téléphonique. Les questions étaient axées sur le comportement, lors du voyage et au retour du voyage, vis à vis de l´utilisation et l´adhésion aux mesures de prévention dont la chimioprophylaxie antipalustre.

### Définitions

Les moyens de prévention individuels du paludisme évoqués dans le 1^er^ questionnaire étaient au nombre de neuf dont six réellement protecteurs (chimioprophylaxie désignée par comprimés, répulsifs, moustiquaire, port de vêtements couvrants, climatisation et diffuseur électrique anti-moustiques) et trois erronés (vaccin, application de crème solaire et application d´antiseptiques suite aux piqûres de moustiques).

En fonction de l´adhésion ou non à ces moyens de prévention, 4 variables catégorielles ont été définies permettant de qualifier le comportement des voyageurs comme «Excellent», «Bon», «Faible» ou «non satisfaisant». Les voyageurs ayant mentionné: la chimioprophylaxie selon les recommandations plus un minimum de trois autres moyens de prévention corrects ont été classés «Excellent»; la chimioprophylaxie selon les recommandations plus un ou deux moyens de prévention corrects ont été classés «Bon»; aucune chimioprophylaxie mais au moins un moyen de prévention correct et pas de réponses erronées ont été classés «Faible»; une ou plusieurs réponses erronées, ont été classés «Non satisafaisant»

L´intension des voyageurs en pré-voyage d´adhérer à ces moyens de prévention et leurs comportements en post voyage vis à vis de ces moyens ont été mesurés par cette classification. La confrontation des réponses des deux questionnaires nous a permis d´apprécier l´évolution des attitudes au cours du séjour en zone impaludée et ainsi l´apport de la séance d´éducation reçue.

**L´analyse statistique:** toutes les données (déjà anonymes) ont été codées et saisies sur un tableau Excel. L´analyse statistique a été faite au moyen du logiciel IBM-SPSS version 23. Les variables catégorielles ont été décrites à travers le calcul de fréquences; et les variables quantitatives, à travers le calcul de moyennes, de médianes et d´écarts types. Les comparaisons de pourcentages ont été effectuées à l´aide du test chi-deux ou du test exact de Fisher et les comparaisons de moyennes à l´aide du test de Student. Pour toutes ces comparaisons, le seuil de signification a été fixé à 0,05.

**Considérations éthiques:** le protocole de l´étude a bénéficié de l´approbation préalable du comité d´éthique bio-médicale de l´IPT (référence: 2017/23/I/SVIAIPT). Le consentement des participants était écrit après lecture d´un document résumant l´étude.

## Résultats

### Profils des voyageurs et du voyage

**Figure 1:**
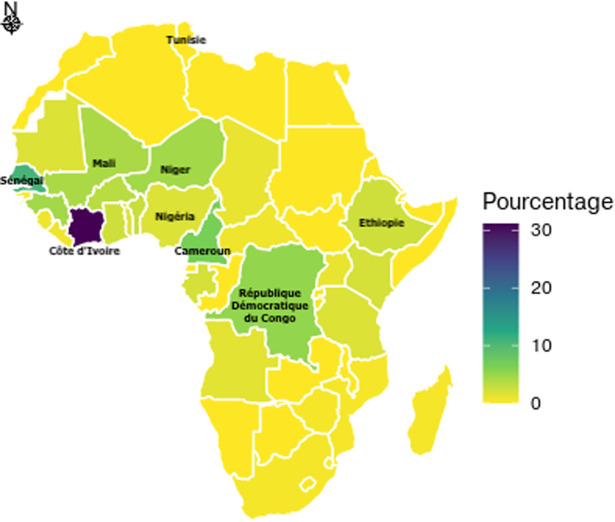
répartition des voyageurs tunisiens en partance vers l´Afrique subsaharienne selon le pays de destination

Au total, 289 voyageurs ont accepté de participer à l´enquête. Ils représentaient 45% (n=289) de nos voyageurs en partance vers un pays d´endémie palustre durant la période de l´étude. Tous ont rempli le questionnaire de pré-voyage et ont bénéficié de l´entretien informatif. L´âge moyen était de 42,3 ans (±12,5). Les hommes représentaient 75,4% (n=218) et les femmes 24,6% (n=71). Le niveau d´instruction était universitaire pour 91,7% (n=265) des participants. Le motif de voyage était professionnel dans 84,4% (n=244) des cas. La destination était l´Afrique SS dans 99% (n=286) des cas. Les pays les plus cités étaient la Côte d´Ivoire (30,1%, n=87) et le Sénégal (10,7%, n=31) (Figure 1). Cent trente neuf (48,1%) voyageurs ont consulté moins de 10 jours avant leur départ. La médiane du délai avant le départ était de 9,5 jours [extrêmes 0 et 72 jours]. La durée de séjour a varié de 2 à 730 jours avec une médiane à 8 jours. Le séjour devait durer moins de 15 jours pour 60,5% (n=175) des voyageurs et plus de 6 mois pour 10% (n=29) d´entre eux. Pour 65,7% (n=190) des voyageurs, il s´agissait d´un premier voyage vers un pays d´endémie palustre. Parmi ceux ayant déjà été en zone d´endémie (n=90), seuls 61% (n=55) avaient pris une chimioprophylaxie antipalustre. Au retour de voyage, 51,2% (n=148) des participants ont répondu à l´entretien téléphonique.

### Perception du risque de transmission du paludisme et connaissance des modalités de transmission

**Figure 2:**
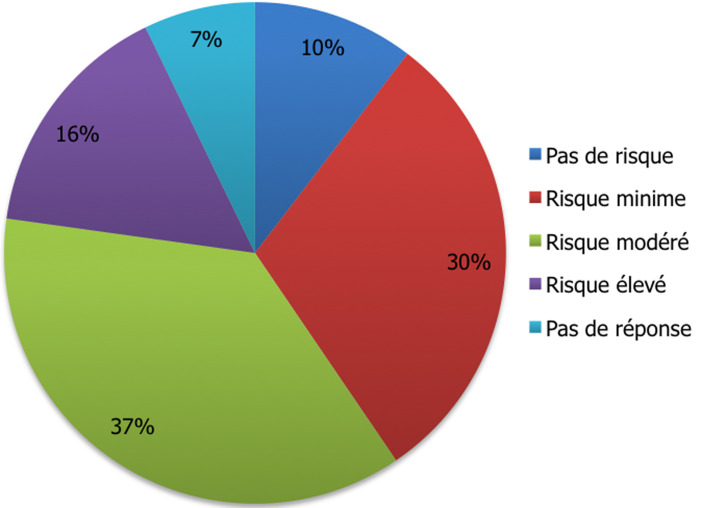
perception par les voyageurs du risque de contracter le paludisme lors du voyage

**Figure 3:**
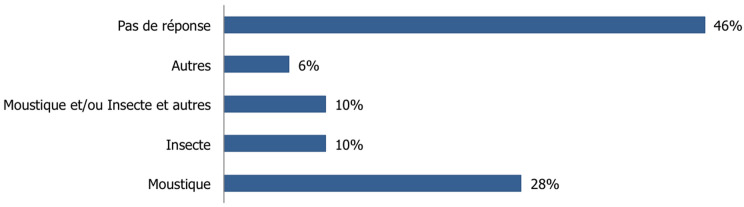
connaissances des voyageurs concernant les modalités de transmission du paludisme

Le risque de contamination par le paludisme était considéré par 66,8% (n=193) des voyageurs comme minime à modéré; alors que 10,4% (n=30) excluaient tout risque et seuls 16,6% (n=48) l´estimaient comme élevé (Figure 2). Parmi les voyageurs, 54% (n=156) ont déclaré connaitre les modalités de transmission du paludisme, dont 28% (n=81) avaient donné des réponses correctes (Figure 3).

### Connaissances des moyens prophylactiques individuels et l´intention d´en utiliser en pré-voyage

**Figure 4:**
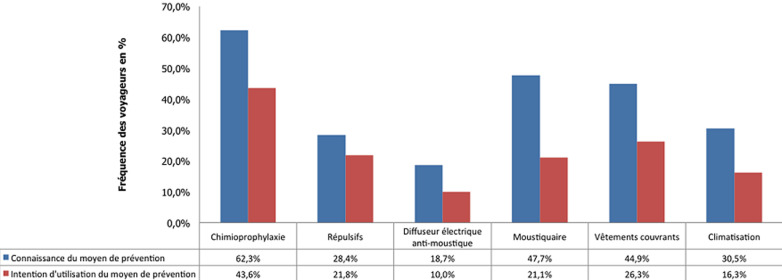
connaissances et intention des voyageurs d’adhérer aux moyens prophylactiques anti-palustres

La chimioprophylaxie a été citée par 62,3% (n=180) des voyageurs. Cependant, 30% (n=54) d´entre eux n´avaient pas l´intention d´en prendre (p < 0,01) (Figure 4). Seuls 28,4% (n=82) des participants ont signalé les répulsifs et 76,8% (n=63) d´entre eux comptaient les appliquer (Figure 4). La moustiquaire a été citée par 47,7% (n=138) des voyageurs et 44,2% (n=61) d´entre eux avaient l´intention d´en utiliser. Le port de vêtements couvrants a été cité par 45% (n=130) des voyageurs dont 58,5% (n=76) comptaient y adhérer (Figure 4). Le diffuseur électrique anti-moustique et la climatisation ont été cités respectivement par 18,7% (n=54) et 30,45% (n=88) des voyageurs (Figure 4). La vaccination a été évoquée à tort par 54,7% (n=158) des voyageurs et 69,6% (n=110) d´entre eux pensaient qu´ils allaient en bénéficier lors de leur consultation. Certains, respectivement 8,9% (n=26) et 22,1% (n=64) de nos voyageurs, ont évoqué l´application de crème solaire et d´antiseptiques locaux (Figure 4). Les sources d´information des voyageurs étaient très diverses. Internet et l´entourage constituaient les principales sources pour 43% (n=125) des voyageurs. Le corps médical n´a été cité que par 11,1% (n=32) des voyageurs et les médias par 12,1% (n=35).

### Evolution en post voyage du comportement des voyageurs vis à vis des moyens de prévention du paludisme

Plus de trois voyageurs sur quatre avaient des réponses initiales de type «non satisfaisant» ou «faible». Les comportements ont évolué dans le bon sens dans le 2^e^ questionnaire. Ainsi, seulement 23,7% (n=35) des voyageurs avaient des attitudes initiales qualifiées de bonne ou excellente contre 64,2% (n=95) au retour (< 0,0001). De nombreuses réponses et comportements classés initialement «bon» ont également progressé vers «excellent».

## Discussion

La mondialisation et l´accroissement des échanges économiques, socioculturels et touristiques, particulièrement avec des pays situés en zones d´endémies infectieuses et parasitaires, exposent de plus en plus de voyageurs à des risques sanitaires [4]. Le paludisme, endémie parasitaire importante en Afrique SS, mais également en Amérique du Sud et en Asie [5], compte parmi les risques majeurs [6,8,9]. En Tunisie, les échanges avec l´Afrique SS ont récemment beaucoup progressé comme en témoigne le lancement de plusieurs lignes aériennes vers la région. L´IPT assure partiellement et de manière initiatique des conseils aux voyageurs exposés à l´occasion de leurs vaccinations internationales. Au cours de ces consultations, il est constaté une méconnaissance manifeste des recommandations et des mesures préventives contre le paludisme. Dans ce contexte, notre enquête avait pour objectif d´évaluer les connaissances du paludisme et de ses modalités de transmission et de prévention afin d´améliorer l´éducation des voyageurs exposés. Les résultats ont confirmé les constatations sus citées en révélant de multiples insuffisances aussi bien des connaissances de la maladie que des comportements au cours des voyages.

La majorité des 289 voyageurs de l´étude étaient des adultes jeunes (âge moyen 42,3±12,5 ans) dont 91,7% avaient un niveau d´instruction universitaire les rendant plus accessibles aux conseils et recommandations. Les séjours étaient plutôt courts (moins de 15 jours dans 60% des cas) ce qui faciliterait l´adhésion aux mesures préventives en en limitant d´éventuelles contraintes et coûts associés. Plus de 50% des voyageurs ont consulté moins de 10 jours avant leur départ, soit un délai relativement court qui entrave une bonne préparation sanitaire du voyage (acquisition de médicaments prophylactiques, de répulsifs et de moustiquaires). En effet, un délai de consultation d´au moins quatre semaines avant le départ est recommandé pour de bons préparatifs des voyages vers ces destinations à risque comme l´a mentionné une enquête réalisée à l´aéroport international JF. Kennedy (NY-Etats-Unis) [10]. De tels délais seraient utiles pour améliorer la perception du risque de contracter le paludisme dont 77,2% de nos voyageurs l´estimaient nul, faible ou modéré. Ils amélioreraient également leurs faibles connaissances des modalités de transmission de la maladie (plus de 50% de réponses fausses) et des moyens prophylactiques recommandés.

Par ailleurs, même quand des connaissances adéquates ont été notées, comme l´utilité de la chimioprophylaxie (62,3%), de la climatisation (48%) et des répulsifs (28,4%), l´intention d´y adhérer était insuffisante, ne dépassant pas les 30% pour la chimioprophylaxie (p < 0,01) et 21% pour les moustiquaires (p < 0,01). Les faibles connaissances s´expliqueraient par la quasi-absence de médiatisation du paludisme en Tunisie (seul 12,1% des voyageurs citent les médias comme source d´information sur le sujet) sachant que la maladie a été éliminée du pays depuis plus de 40 ans et que les nouvelles générations ne l´ont jamais connue. De plus, les destinations à risque sont nouvelles pour les Tunisiens habitués jusque là à se rendre plutôt en Europe, au Maghreb et au Moyen-Orient pas ou peu touchés par la parasitose. Le manque de connaissances est aussi lié à la dispersion et au caractère épisodique des informations disponibles, souvent restreintes au personnel navigant, aux sportifs engagés dans des compétitions continentales et aux agents de quelques prestataires impliqués en Afrique SS. L´absence de consultations spécialisées de médecine de voyages (MV), à l´instar des pays occidentaux [8], accentue les résultats inquiétants obtenus. La promotion de telles consultations améliorerait certainement l´accessibilité des informations utiles ainsi que leur qualité [11,12].

La faible adhésion à la chimioprophylaxie, déjà rapportée dans d´autres séries tunisiennes [1], est un problème majeur, malgré que certaines molécules sont fournies gratuitement. Un tel constat est mentionné à l´échelle internationale [13]. Ainsi Chen *et al*. signalent que 89% des cas de paludisme à *P. falciparum* survenus lors de voyages professionnels sont observés chez des sujets n´ayant pas pris de chimioprophylaxie [11]. La non-adhésion à la chimioprophylaxie pourrait par ailleurs s´expliquer par la mauvaise réputation sur les réseaux sociaux de certains produits [13], les effets indésirables vécus lors de prises antérieures et le coût élevé de certaines molécules [10,14]. Elle pourrait aussi trouver origine dans la diversité et la variabilité des recommandations selon les pays et parfois un manque de clarté de certaines d´entre elles [15].

Si le questionnaire de pré-voyage a mis en évidence des connaissances insuffisantes et des intentions d´attitude sur place plutôt inquiétantes, celui de post-voyage a permis de constater une évolution dans le bon sens des paramètres évalués. Ainsi, les attitudes considérées « bonnes » ou « excellentes » en terme de prévention sont passées de 23,7% à 64,2% des voyageurs (p < 0,001). Ce résultat s´explique sans doute par l´entretien médical pédagogique accordé aux voyageurs avant leurs voyages. Le niveau d´instruction élevé de ces derniers les ayant certainement rendu réceptifs aux messages. Le fait d´insister sur les risques encourus a probablement fixé leur attention et encouragé leur adhésion. En effet, cette perception du risque était particulièrement faible chez nos voyageurs avec seulement 15,6% qui le considéraient important contre 73% d´Américains voyageant vers les mêmes destinations [10]. La perception du risque est une étape fondamentale pour atteindre les résultats escomptés. La polyclinique médicale de Lausanne a développé pour cela un outil d´aide à la décision qui implique le voyageur afin de mieux le sensibiliser [14]. Dans cette lignée, un outil italien nommé «TRiP questionnaire» vise à évaluer cette perception du risque encouru et d´en déterminer les facteurs [16].

Notre étude a révélé des insuffisances concernant la connaissance et la prévention du paludisme par les voyageurs en partance vers les zones d´endémie palustre. Les données recueillies permettront de corriger les manquements et d´améliorer les performances de la stratégie d´information et de prévention. La limitation de l´incidence des cas importés de la maladie, en plus de ses impacts sanitaire et économique directs [17], permettrait également de mieux contrôler toute éventuelle circulation autochtone des plasmodiums comme déjà rapporté dans d´autres pays méditerranéens [18]. Les centres de MV devraient également être promus afin de délivrer les conseils et prescriptions utiles concernant le paludisme, mais aussi d´autres pathologies émergentes comme certaines arboviroses (Zika, Chkungunyia, Dengue) dont le vecteur vient d´être identifié en Tunisie [8,19].

## Conclusion

Les connaissances du paludisme, de sa transmission et de sa prévention se sont révélées faibles chez le voyageur tunisien en partance vers les pays d´endémie. Une consultation médicale de pré-voyage a cependant permis de les améliorer significativement. Notre stratégie d´information sur la maladie devrait être optimisée en conséquence notamment par la promotion de centres spécialisés de MV.

### Etat des connaissances sur le sujet

- Les zones à risque de transmission de paludisme, les modalités de transmission et les moyens de prévention sont parfaitement connus;
- L´épidémiologie du paludisme en Tunisie, en particulier l´incidence annuelle des cas ainsi que leur profil sont aussi bien connus.

### Contribution de notre étude à la connaissance

- Estimation du niveau de connaissances des voyageurs tunisiens quand au paludisme, sa transmission et ses mesures prophylactiques;
- Evaluation du comportement des voyageurs au cours de leur séjour en zone d´endémie palustre; évaluation de l´adhésion des voyageurs aux messages d´éducation sanitaire quand à la prévention du paludisme au cours de séjours à risque;
- Collecter des données chiffrées pour une actualisation de la stratégie nationale en termes de prévention du paludisme.

## Remerciements

Mme Hayet Moussa pour la révision du questionnaire de l´enquête. Mlle Karima Hechmi qui a assuré la logistique de l´enquête lors de sa réalisation. Mr Oussema Souiai pour avoir participé à la révision du document.

## References

[1] Aoun K, Siala E, Tchibkere D, Ben Abdallah R, Zallagua N, Chahed MK (2010). Imported malaria in Tunisia: consequences on the risk of resurgence of the disease. Med Trop Rev Corps Sante Colon.

[2] Siala E, Gamara D, Kallel K, Daaboub J, Zouiten F, Houzé S (2015). Airport malaria: report of four cases in Tunisia. Malar J.

[3] Tabbabi A, Boussès P, Rhim A, Brengues C, Daaboub J, Ben-Alaya-Bouafif N (2015). Larval habitats characterization and species composition of Anopheles mosquitoes in Tunisia, with particular attention to Anopheles maculipennis complex. Am J Trop Med Hyg.

[4] Buss I, Genton B, D´Acremont V (2020). Aetiology of fever in returning travelers and migrants: a systematic review and meta-analysis. J Travel Med.

[5] Thellier M, Simard F, Musset L, Cot M, Velut G, Kendjo E (2020). Changes in malaria epidemiology in France and worldwide, 2000-201 Médecine Mal Infect.

[6] Pradines B, Thellier M, Kendjo E, Houze S (2019). [Epidemiology of imported malaria to France]. Rev Prat.

[7] World Health Organization, éditeur (2016). International health regulations (2005).

[8] Chen LH, Wilson ME (2002). Recent Advances and New Challenges in Travel Medicine. Curr Infect Dis Rep.

[9] Talbot EA, Chen LH, Sanford C, McCarthy A, Leder K, Research Committee of International Society of Travel Medicine (2010). Travel medicine research priorities: establishing an evidence base. J Travel Med.

[10] Hamer DH, Connor BA (2004). Travel Health Knowledge, Attitudes and Practices among United States Travelers. J Travel Med.

[11] Chen LH, Leder K, Barbre KA, Schlagenhauf P, Libman M, Keystone J (2018). Business travel-associated illness: a GeoSentinel analysis. J Travel Med.

[12] Mahadevan SV, Strehlow MC (2017). Preparing for International Travel and Global Medical Care. Emerg Med Clin North Am.

[13] Dupouy-Camet J, Yera H, Tourte-Schaeffer C (2003). Problems in prescribing malaria chemoprophylaxis for travelers. Fundam Clin Pharmacol.

[14] Auer R, Voumard R, Benaroyo L, Genton B (2015). Communication du risqueen médecine des voyages. Rev Med Suisse.

[15] Schlagenhauf P, Petersen E (2008). Malaria Chemoprophylaxis: Strategies for Risk Groups. Clin Microbiol Rev.

[16] Tardivo S, Zenere A, Moretti F, Marchiori F, Berti D, Migliorini M (2020). The Traveller´s Risk Perception (TRiP) questionnaire: pre-travel assessment and post-travel changes. Int Health.

[17] Khuu D, Eberhard ML, Bristow BN, Javanbakht M, Ash LR, Shafir SC (2019). Economic impact of malaria-related hospitalizations in the United States, 2000-2014. J Infect Public Health.

[18] Odolini S, Gautret P, Parola P (2012). Epidemiology of imported malaria in the mediterranean region. Mediterr J Hematol Infect Dis.

[19] Bohers C, Mousson L, Madec Y, Vazeille M, Rhim A, M´ghirbi Y (2020). The recently introduced Aedes albopictus in Tunisia has the potential to transmit chikungunya, dengue and Zika viruses. PLoS Negl Trop Dis [Internet].

